# Heteronanostructural metal oxide-based gas microsensors

**DOI:** 10.1038/s41378-022-00410-1

**Published:** 2022-07-28

**Authors:** Lin Liu, Yingyi Wang, Yinhang Liu, Shuqi Wang, Tie Li, Simin Feng, Sujie Qin, Ting Zhang

**Affiliations:** 1 i-Lab, Key Laboratory of Multifunctional Nanomaterials and Smart Systems, Suzhou Institute of Nano-Tech and Nano-Bionics (SINANO), Chinese Academy of Sciences (CAS), Suzhou, Jiangsu China; 2 Department of Health and Environmental Sciences, Xi’an Jiaotong-Liverpool University, Suzhou, Jiangsu China; 3 Department of Nano Science and Nano Technology Institute, University of Science and Technology of China, Suzhou, Jiangsu China; 4 Nano-X, Suzhou Institute of Nano-Tech and Nano-Bionics (SINANO), Chinese Academy of Sciences (CAS), Suzhou, Jiangsu China; 5 School of Nano-Tech and Nano-Bionics, University of Science and Technology of China, Hefei, Anhui PR China; 6 Gusu Laboratory of Materials, Suzhou, Jiangsu PR China; 7 Center for Excellence in Brain Science and Intelligence Technology, Chinese Academy of Sciences, Shanghai, PR China

**Keywords:** Nanosensors, Structural properties

## Abstract

The development of high-performance, portable and miniaturized gas sensors has aroused increasing interest in the fields of environmental monitoring, security, medical diagnosis, and agriculture. Among different detection tools, metal oxide semiconductor (MOS)-based chemiresistive gas sensors are the most popular choice in commercial applications and have the advantages of high stability, low cost, and high sensitivity. One of the most important ways to further enhance the sensor performance is to construct MOS-based nanoscale heterojunctions (heteronanostructural MOSs) from MOS nanomaterials. However, the sensing mechanism of heteronanostructural MOS-based sensors is different from that of single MOS-based gas sensors in that it is fairly complex. The performance of the sensors is influenced by various parameters, including the physical and chemical properties of the sensing materials (*e.g*., grain size, density of defects, and oxygen vacancies of materials), working temperatures, and device structures. This review introduces several concepts in the design of high-performance gas sensors by analyzing the sensing mechanism of heteronanostructural MOS-based sensors. In addition, the influence of the geometric device structure determined by the interconnection between the sensing materials and the working electrodes is discussed. To systematically investigate the sensing behavior of the sensor, the general sensing mechanism of three typical types of geometric device structures based on different heteronanostructural materials are introduced and discussed in this review. This review will provide guidelines for readers studying the sensing mechanism of gas sensors and designing high-performance gas sensors in the future.

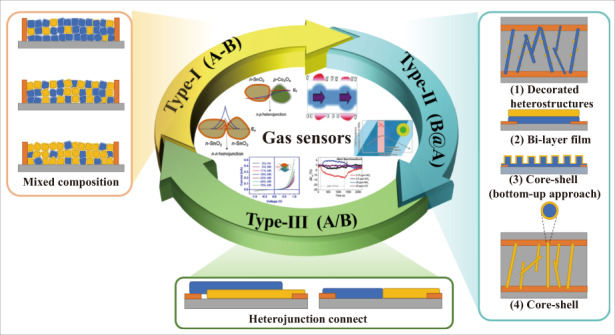

## Introduction

Air pollution is becoming a growing concern and a serious worldwide environmental problem that threatens the wellbeing of humans and organisms. Inhalation of gas pollutants can cause many health problems, such as respiratory disease, lung cancer, leukemia, and even early death^1–4^. It is reported that from 2012 to 2016, millions of people died from air pollution, and billions of people face poor air quality every year^5^. Therefore, it is important to develop portable and miniaturized gas sensors that can provide real-time feedback and high sensing performance (*e.g*., sensitivity, selectivity, stability, and response and recovery time). In addition to environmental monitoring, gas sensors also play a crucial role in security^6–8^, medical diagnosis^9,10^, aquaculture^11^, and other fields^12^.

To date, several types of portable gas sensors based on different sensing mechanisms, such as optical^13–18^, electrochemical^19–22^, and chemiresistive sensors^23,24^, have been made available. Among them, metal oxide semiconductor (MOS)-based chemiresistive sensors are the most popular in commercial applications due to their high stability and low cost^25,26^. The concentration of pollutants can be obtained by simply detecting changes in the resistance of MOSs. At the beginning of the 1960s, the first chemiresistive gas sensor based on ZnO film was reported, and it aroused great interest in the field of gas sensing^27,28^. To date, many different MOSs have been used as gas sensing materials, and they can be divided into two classes according to their physical properties: n-type MOSs where electrons are the majority charge carriers and p-type MOSs where holes are the majority charge carriers. Normally, p-type MOSs are less popular than n-type MOSs because the sensing response of a p-type MOS (*S*_*p*_) is proportional to the square root of an n-type MOS ($S_p = \sqrt {S_n}$) under the same presumptions (*e.g*., same morphological configurations and same band bending changes in the air)^29,30^. However, the practical applications of single MOS-based sensors still encounter some issues, such as insufficient detection limit and poor sensitivity and selectivity. The selectivity issue can be addressed to some degree by constructing sensor arrays, known as “electronic noses”, and by combining computational analysis algorithms such as learning vector quantization (LVQ), principal component analysis (PCA), and partial least squares (PLS) analysis^31–35^. In addition, fabrication of low-dimensional MOSs^32,36–39^ (*e.g*., one-dimensional (1D), 0D and 2D nanomaterials) and modification of backbone MOSs with other nanomaterials (*e.g*., MOSs^40–42^, noble metal nanoparticles (NPs)^43,44^, carbon nanomaterials^45,46^, and conducting polymers^47,48^) to construct nanoscale heterojunctions (*i.e*., heteronanostructural MOSs) are the other preferred approaches to tackle the abovementioned issues. Compared with conventional thick MOS films, low-dimensional MOSs with large specific surface areas could provide more activation sites for gas adsorption and facilitate gas diffusion^36,37,49^. In addition, the design of MOS-based heteronanostructures can further modulate carrier transport at the heterointerface, leading to larger resistance changes due to the different working functions^50–52^. Moreover, some chemical effects (*e.g*., catalytic activity and synergistic surface reactions) originating from designing MOS heteronnanostructures could also improve the sensor performance^50,53,54^. Although the design and construction of MOS-based heteronanostructures would be a promising approach to enhance the sensor performance, current chemiresistive sensors often use a trial-and-error type approach, which is time-consuming and inefficient. Therefore, it is important to understand the sensing mechanism of MOS-based gas sensors, as it can provide a guideline for the directional design of high-performance sensors.

In recent years, MOS-based gas sensors have undergone rapid development, and some review papers about MOS nanostructures^55–57^, room-temperature gas sensors^58,59^, specific MOS sensing materials^60–62^, and specific gas sensors^63^ have been reported. Other reviews have focused on elucidating the sensing mechanism of gas sensors according to the intrinsic physical and chemical properties of the MOSs, including the role of oxygen vacancies^64^, the role of heteronanostructure^55,65^, and charge transfer at the heterointerface^66^. Moreover, the sensor performance is also influenced by various other parameters, including heterostructure, grain size, operation temperature, defect density, oxygen vacancy, and even the exposed crystal facet of the sensing materials^25,67–73^. However, the geometric device structure determined by the interconnection between the sensing materials and the working electrodes, which is seldom mentioned, can also significantly affect the sensing behavior of the sensor^74–76^ (more details are provided in Section 3). For example, Kumar et al.^77^ reported two gas sensors based on the same materials (*e.g*., TiO_2_@NiO and NiO@TiO_2_ bilayer-based gas sensors) and observed different resistance changes upon NH_3_ gases due to the different geometric structures of the devices. Therefore, it is important to consider the device structure when analyzing the gas sensing mechanism. In this review, the authors focus on the sensing mechanism based on different heteronanostructures of MOSs and the structures of the devices. We believe that this review could provide guidelines for readers who wish to understand and analyze the gas sensing mechanism, and it may facilitate the design of high-performance gas sensors in the future.

## Basic sensing mechanism of a single material and enhancement sensing mechanism of a MOS heterostructure

**Fig. 1:**
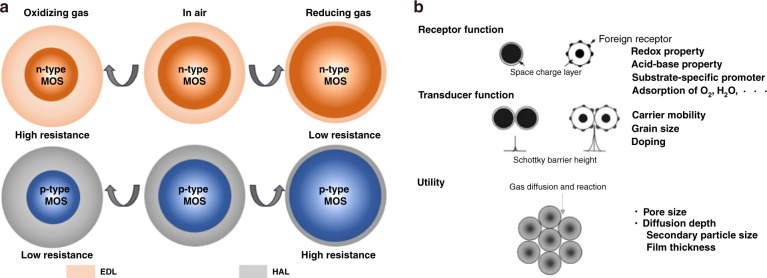
Basic sensing mechanism of MOS-based gas sensors. **a** Basic sensing mechanism of n- and p-type MOSs toward reducing and oxidizing gases; **b** Key factors and physicochemical or material properties involved in semiconductor gas sensors^89^

Figure 1a illustrates the basic gas sensing mechanism model based on a single MOS. When the temperature increases, the adsorption of oxygen molecules (O_2_) on the surface of MOS attracts electrons from MOS and form anionic species (such as O^2−^ and O^−^). Then, an electron-depletion layer (EDL) for an n-type MOS or a hole accumulation layer (HAL) for a p-type MOS is formed at the surface of the MOS^15,23,78^. The interaction between O_2_ and MOSs leads to upward bending of the MOS conduction band at the surface and forms a potential barrier. Later, when the sensor is exposed to target gases, the gases adsorbed on the surface of the MOS react with ionic oxygen species by attracting electrons (oxidizing gases) or donating electrons (reducing gases)^79,80^. The transfer of the electrons between the target gases and MOS can regulate the width of EDL or HAL^30,81^, resulting in a change in the overall resistance for the MOS-based sensors. For example, for reducing gases, the electrons will transfer from the reducing gases to the n-type MOS, leading to a decrease in EDL and a decrease in resistance, which is named n-type sensing behavior. In contrast, p-type sensing behavior is defined when a p-type MOS is exposed to the reducing gases, HAL will shrink and the resistance will increase due to the donated electrons. For oxidizing gases, the sensor response is opposite to that of reducing gases.

Except for the basic sensing mechanism, the gas sensing mechanisms involved in practical gas sensors are fairly complex. For instance, the practical use of gas sensors should meet many requirements (*e.g*., sensitivity, selectivity, and stability) depending on the users’ needs. These requirements are closely correlated with the physical and chemical properties of the sensing materials. For example, Xu et al.^71^ demonstrated that a SnO_2_-based sensor reached the highest sensitivity when the crystal diameter (*d*) was comparable to or less than twice the Debye length (*λ*_D_) of SnO_2_^71^. When *d* ≤ 2*λ*_*D*_, the SnO_2_ is completely depleted after adsorption of O_2_ molecules, and the sensing response to the reducing gases is maximized. In addition, various other parameters can also influence the sensor performance, including working temperatures, crystal defects, and even the exposed crystal facets of the sensing materials^25,67–73^. Specifically, the influence of the working temperatures is ascribed to the possible competition between adsorption and desorption rates of the target gases and the surface reactivity between the adsorbed gas molecules and oxygen species^4,82^. The effect of crystal defects is greatly related to the content of oxygen vacancies^83,84^. The sensor performance can also be influenced by the exposed crystal facets due to their different reactivities^67,85–87^. The exposed crystal facets with lower density reveal more uncoordinated metal cations with higher energy that facilitate surface adsorption and reactivity^88^. Several main factors and relevant enhancement sensing mechanisms are listed in Table 1. Therefore, by tuning these material parameters, the sensing performance can be improved, and it is crucial for determining the key factors that influence the sensor performance.

**Table 1:** Summary of the main factors that influence the sensor performance and relevant enhancement sensing mechanism

Main factors	Materials	Target gases	Response (ppm/value)	Enhancement sensing mechanism	Ref
Grain size	SnO_2_	Butane	8219.2/8.09^c^	Smaller grain size induced large specific surface area, increased grain boundaries and volume depletion (d ~ 2*λ*_*d*_).	^186^
	Zeolite/SnO_2_	Formaldehyde	10/11^c^		^187^
	ZnO	H_2_S	0.05/0.142^a^		^188^
	Pd@ZnO	H_2_	10000/~8.5^a^		^170^
	In_2_O_3_	Formaldehyde	50/~14^c^		^189^
	SnO_2_	H_2_S	5/13000^c^	Relative larger grain size in fine grain films (d=0-20 nm) promotes gas diffusion.	^68^
	Fe_2_O_3_	Ethanol	100/14.5^c^		^190^
	SnO_2_	CO	50/4.5^c^		^191^
Effects of Heterojunctions	PdO@ZnO	Ethanol	100/35.4^c^	Heterojunction induces more active site; catalytic effect; modulation of conduction channel and carrier transportation etc.	^192^
	Fe_2_O_3_@NiO	Acetone	100/290^b^		^193^
	ZnO-Co_3_O_4_	Triethylamine	50/67.8^c^		^194^
	ZnO-SnO_2_-RGO	NO_2_	5/141.0^d^		^195^
	NiO@SnO_2_	NO_2_	100/~4.4^b^		^196^
	SnO_2_-Co_3_O_4_	Toluene	1/18.7^c^		^98^
	CuO-In_2_O_3_	H_2_S	5/229.3^c^		^105^
	ZnO-Co_3_O_4_	Ethanol	1000/106^b^		^106^
Density of defect	ZnO	Acetone	5/~56^b^	Increased donor defects provide more free electrons for forming active oxygen species.	^83^
	ZnO	Ethanol	200/193.7^b^		^197^
	Co/ZnO	Triethylamine	50/1020^c^		^198^
	ZnO	Ethanol	100/47^c^		^199^
	ZnO	Formaldehyde	200/227.4^c^		^200^
	TiO_2_	H_2_	10/58^d^	Increased oxygen vacancies provide more oxygen adsorption sites.	^201^
	Ce-Fe_2_O_3_	Acetone	100/26.3^c^		^202^
	W_18_O_49_@ PANI	NH_3_	100/50^b^		^203^
	SnO_2_	H_2_	0.1/1.25^c^		^204^
	Mo-SnO_2_	Ethanol	100/46.8^b^		^205^
	Tb- SnO_2_	Ethanol	100/53.6^c^		^206^
	Sb- SnO_2_/ZnO	NO_2_	1/9.5^a^		^207^
	ZnO	CO	0.45/24.9^c^		^49^
	SnO_2_	Ethanol	100/24.9^c^		^208^
	Bi_2_MoO_6_	NH_3_	5/53.97^b^		^209^
Exposed crystal facet	In_2_O_3_	Ethanol	1000/610^c^	The optimized exposed crystal facet could provide more oxygen vacancies and dangling bonds.	^210^
	ZnO	Ethanol	50/80^c^		^211^
	SnO_2_	Formaldehyde	200/207.7^c^		^212^
	CeO_2_	Dimethylamine	100/142^c^		^87^
	Co_3_O_4_	NO_2_	0.3/0.16^d^	The optimized exposed crystal facet could provide more lattice oxygen to interact with gases.	^213^
	SnO_2_	Alkenes	50/40^c^		^214^
	Au@SnO_2_	Acetylene	100/77.5^b^	The optimized exposed crystal facet exhibits higher adsorption energy.	^215^

a: (R_g_-R_a_)/R_a_ b: R_g_/R_a_ c: R_a_/R_g_ d: (R_g_-R_a_)/R_a_*100

Yamazoe^89^ and Shimanoe et al.^68,71^ performed many works to study the theoretical sensing mechanism of the sensors and proposed three independent key factors that can affect the performance of the sensors, specifically the receptor function, transducer function, and utility (Fig. 1b). Receptor function means the ability of the MOS surface to interact with gas molecules. This function is strongly related to the chemical properties of MOS and can be largely improved by introducing foreign receptors (*e.g*., metal NPs and other MOSs). Transducer function refers to the ability to convert reactions between the gases and MOS surface into electrical signals, and this function is dominated by the grain boundary of MOSs. Therefore, the transducer function is significantly influenced by the grain size of MOSs and the density of foreign receptors. Katoch et al.^90^ reported that reducing the grain size of ZnO-SnO_2_ nanofibrils leads to the formation of a large number of heterojunctions and improves the sensor sensitivity, which is consistent with the transducer function. Wang et al.^91^ compared different Zn_2_GeO_4_ grain sizes and demonstrated that the sensor sensitivity increased by 6.5 times after grain boundaries were introduced. Utility is another key factor in the performance of the sensors that describes the accessibility of interior MOSs to gases. If the gas molecules cannot access and react with the interior MOSs, the sensor responsivity is reduced. Utility is closely related to the diffusion depth of a specific gas, which is dependent on the pore size of the sensing materials. Sakai et al.^92^ simulated the sensing response of sensors to combustion gases with respect to different diffusion depths of gases inside the sensing films and found that both the molecular weight of the gas and the pore radius of the sensing film can affect the sensor response. Through the above discussions, it is demonstrated that high-performance gas sensors can be designed by balancing and optimizing the receptor function, transducer function and utility^93^.

The abovementioned works have elucidated the basic sensing mechanism of a single MOS and discussed several factors that impact the MOS performance. In addition to these factors, gas sensors based on heterostructures can further improve the sensor performance by greatly enhancing the transducer function and receptor function^93^. In addition, heteronanostructures can further improve the sensor performance by enhancing catalytic reactions, modulating charge transport, and providing more adsorption sites^94^. To date, numerous MOS heteronanostructure-based gas sensors have been studied to discuss the enhancement sensing mechanism^95–97^. Miller et al.^55^ summarized several of the most likely mechanisms responsible for enhancing the sensing performance of heteronanostructures, including surface-dependent, interface-dependent, and structure-dependent. Among them, the interface-dependent enhancement mechanism is too complicated to cover all interface interactions by one theory, as various heteronanostructural material-based sensors are available (*e.g*., n-n heterojunctions, p-n heterojunctions, p-p heterojunctions, and Schottky junctions). Generally, sensors based on heteronanostructural MOSs always involve more than two or three enhancement sensing mechanisms^98–100^. The synergistic effects of these enhancement mechanisms can amplify the reception and transduction of the sensor signal^101^. Therefore, understanding the sensing mechanism of sensors based on heteronanostructural materials is essential and can guide researchers in the bottom-up design of gas sensors according to their demands. In addition, the geometric device structure can also significantly affect the sensing behavior of the sensor^74–76^. To systematically analyze the sensing behavior of the sensor, the sensing mechanism of three types of device structures based on different heteronanostructural materials will be introduced and discussed in the following sections.

## Three typical device structures and relevant sensing mechanisms

With the rapid development of MOS-based gas sensors, various heteronanostructural MOSs have been proposed. The charge transfer at the heterointerface depends on the different Fermi levels (E_f_) of the components. At the heterointerface, the electrons move from one side with higher E_f_ to the other with lower E_f_ until their Fermi levels reach equilibrium, and vice versa for holes. Then, the carriers at the heterointerface are depleted and form a depletion layer. Once the sensor is exposed to target gases, the carrier concentration of heteronanostructural MOSs changes, as does the potential barrier height, which amplifies the detection signal. In addition, the various approaches to fabricating heteronanostructures lead to varied interconnections between the materials and electrodes, which produces different geometric device structures and leads to different sensing mechanisms. In this review, we propose three types of geometric device structures and discuss the sensing mechanism of each structure.

### Definition of three types of device structures

**Fig. 2:**
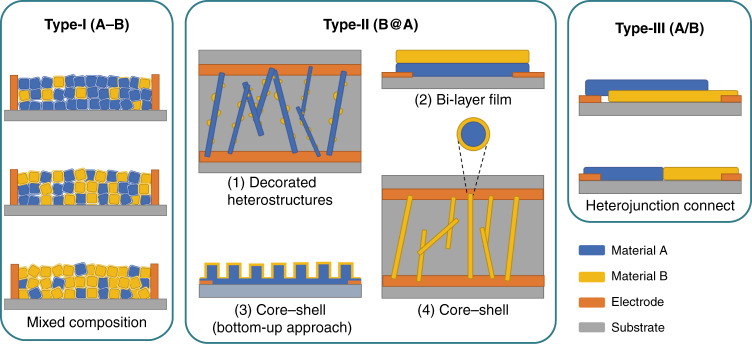
Three types of device structures. A hyphen between compounds (*e.g*., “SnO_2_-NiO”) indicates that the two constituents are simply mixed (type-I). An “@” between two compounds (*e.g*., “SnO_2_@NiO”) indicates that the backbone material (NiO) is decorated with SnO_2_, which is used in the type-II sensor structure. A forward slash (*e.g*., “NiO/SnO_2_”) represents the type-III sensor structure

Although the heterojunction plays a very important role in gas sensing performance, the geometric device structure of the whole sensor can also significantly affect the sensing behavior because the position of the conduction channel of the sensor greatly depends on the geometric device structure. Here, three typical types of geometric device structures based on MOS heterojunctions are discussed and shown in Fig. 2. In the first type, two MOS compounds are randomly distributed between two electrodes, and the position of the conduction channel is determined by the major MOS. In the second type, heteronanostructures are formed from different MOSs, while only one MOS is connected to the electrodes, and the conduction channel is normally located inside one MOS, which is directly connected to the electrodes. In the third type, two materials are connected to the two electrodes separately, and the device is channeled by the formed heterojunction between the two materials.

### Sensing Mechanism of the Type-I Sensor Structure

For MOS composite-based gas sensors, two kinds of MOSs are randomly distributed between the electrodes. Many fabrication methods have been developed to obtain MOS composites, including sol-gel, coprecipitation, hydrothermal, electrospinning, and mechanical mixing methods^98,102–104^. Recently, metal–organic frameworks (MOFs), a class of porous crystalline framework-structured materials, which consist of a metal center and an organic linker, have been used as a template to fabricate porous MOS composites^105–108^. Notably, even though the composition percentages of MOS composites are the same, the sensing performance can be quite different when different fabrication processes are used^109,110^. For instance, Gao et al.^109^ prepared two sensors based on MoO_3_-SnO_2_ composites with the same atomic ratio (Mo:Sn = 1:1.9) and found that different fabrication methods led to different responsivities. Shaposhnik et al.^110^ reported that the response of coprecipitated SnO_2_-TiO_2_ to H_2_ gases differs from that of the material formed by mechanical mixing, even though the Sn/Ti ratio was the same. This difference occurred because the interconnections between the MOSs and the crystallite size of the MOSs differ with various synthesis methods^109,110^. When the size and shape of the grains are uniform in terms of donor density and semiconductor type, the response results should remain the same if the contact geometry is unchanged^110^. Staerz et al.^111^ reported that the sensing performance of SnO_2_-Cr_2_O_3_ core-shell nanofibers (CSNs) and crushed SnO_2_-Cr_2_O_3_ CSNs are practically identical, indicating that the nanofiber morphology shows no advantages if the characteristics of the samples remain unchanged.

**Fig. 3:**
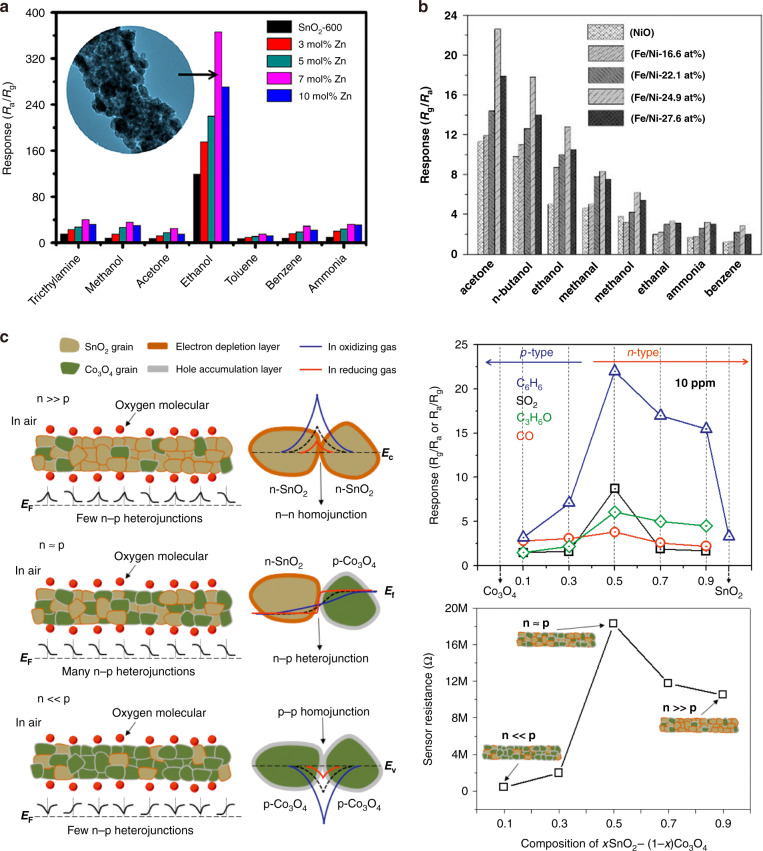
The influence of heterojunction number. **a** SEM image of a SnO_2_-ZnO fiber (7 mol% Zn) and responses of the sensors to various 100 ppm gases at 260 °C;^54^ **b** responses of the sensors based on pure NiO and NiO-NiFe_2_O_4_ composites to 50 ppm different gases at 260 °C;^114^ (**c**) schematic diagram of the number of junctions in the xSnO_2_-(1−x)Co_3_O_4_ compositions and relevant resistance and sensing response of xSnO_2_-(1−x)Co_3_O_4_ compositions to 10 ppm CO, acetone, C_6_H_6_, and SO_2_ gases at 350 °C by varying the Sn/Co molar ratio^98^

In addition to different fabrication methods, the semiconducting type of two different MOSs can also influence the sensing behavior of the sensors. This can be further classified into two categories depending on whether the two MOSs are of the same type of semiconductors (n-n or p-p composite) or different types (p-n composite). When the types of MOS composite-based gas sensors are the same, the sensing response behavior remains unchanged by varying the molar ratio of the two MOSs, while the sensitivity of the sensor changes as the number of n-n or p-p heterojunctions is different^102^. When one component dominates in the composites (*e.g*., 0.9 ZnO-0.1 SnO_2_ or 0.1 ZnO-0.9 SnO_2_), the conduction channel is determined by the major MOS, which is called the homojunction conduction channel^92^. When the proportion of two components is comparable, the conduction channel is believed to be dominated by the heterojunction^98,102^. Yamazoe et al.^112,113^ reported that the heterocontact regions of two components could dramatically enhance the sensitivity of the sensor because the heterojunction potential barrier formed due to the different work functions of the components can efficiently tune the drift mobility of electrons when the sensor is exposed to different ambient gases^112,113^. Figure 3a shows that the sensors based on fibrous SnO_2_-ZnO hierarchical structures with different ZnO contents (0 to 10 mol% Zn) can selectively detect ethanol^54^. Among them, the sensor based on the SnO_2_-ZnO fiber (7 mol% Zn) exhibited the highest sensitivity due to the formation of a large number of heterojunctions and an increase in the specific surface area, which increased the transducer function and enhanced the sensitivity^90^. However, by further increasing the ZnO component to 10 mol%, the microstructure of the SnO_2_-ZnO composites might wrap up the surface activation sites and reduce the sensing response^85^. A similar tendency can be observed for sensors based on NiO-NiFe_2_O_4_ p-p heterojunction composites with different Fe/Ni ratios (Fig. 3b)^114^.

For the case of p-n MOS composites, they exhibit different sensing behaviors depending on the atomic ratio of MOSs^115^. Generally, the sensing behavior of MOS composites greatly depends on which MOS acts as the dominating conduction channel of the sensor. Therefore, it is very important to characterize the percentage composition and the nanostructure of the composites. Kim et al.^98^ verified this conclusion by synthesizing a series of composite xSnO_2_-(1−x)Co_3_O_4_ nanofibers via an electrospinning method and studying their sensing performance. They observed that the sensing behavior of sensors based on SnO_2_-Co_3_O_4_ composites transits from n-type to p-type sensing behavior by reducing the SnO_2_ percentage (Fig. 3c)^98^. Moreover, compared with homojunction-dominated sensors (*e.g*., SnO_2_-rich or Co_3_O_4_-rich sensors), the heterojunction-dominated sensor (0.5 SnO_2_-0.5 Co_3_O_4_-based) exhibits the highest sensing response to C_6_H_6_. The intrinsic high resistance of 0.5 SnO_2_-0.5 Co_3_O_4_-based sensor and its higher ability to modulate the total resistance of the sensor contribute to its supreme sensitivity to C_6_H_6_. Moreover, defects originating from lattice mismatch form at the SnO_2_-Co_3_O_4_ heterointerfaces, which can provide preferential adsorption sites for gas molecules, lead to an enhanced sensing response^109,116^.

**Fig. 4:**
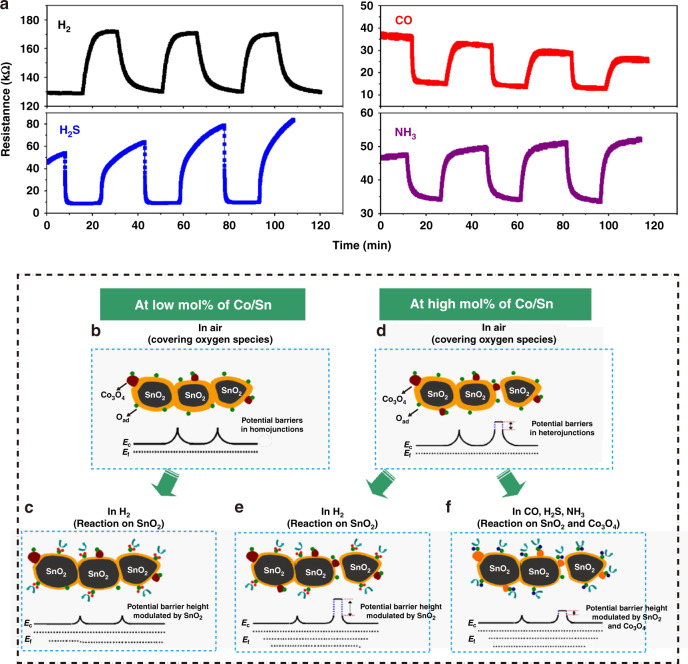
The influence of the chemical properties of MOSs. **a** Dynamic resistance curves of Co/Sn-10% to the typical reducing gases of H_2_, CO, NH_3_, and H_2_S; **b**, **c** the sensing mechanism diagrams of Co_3_O_4_/SnO_2_ composites to H_2_ at low mor% of Co/Sn; **d**–**f** the sensing mechanism diagrams of Co_3_O_4_/SnO_2_ composites to H_2_ and CO, H_2_S, and NH_3_ at high mor% of Co/Sn

In addition to the semiconducting type of MOSs, the sensing behavior of MOS composites can also be modulated by the chemical properties of the MOS^117^. Huo et al.^117^ used a simple soak calcination method to fabricate Co_3_O_4_-SnO_2_ composites and observed that when the Co/Sn molar ratio was 10%, the sensor exhibited a p-type sensing response to H_2_, while it demonstrated an n-type sensing response to CO, H_2_S, and NH_3_ gases, as shown in Fig. 4a^117^. With a low Co/Sn ratio, many homojunctions form at the SnO_2_-SnO_2_ nanograin boundaries and exhibit an n-type sensing response to H_2_ (Fig. 4b, c)^115^. By increasing the Co/Sn ratio to 10 mol%, many Co_3_O_4_-SnO_2_ heterojunctions simultaneously form instead of the SnO_2_-SnO_2_ homojunction (Fig. 4d). Since Co_3_O_4_ is inactive to H_2_ while SnO_2_ is highly reactive to H_2_, the reaction between H_2_ and ionic oxygen species mainly occurs on the surface of SnO_2_^117^. Therefore, the electrons are transferred to SnO_2_ and shift the E_f_ of SnO_2_ toward the conduction band, while the E_f_ of Co_3_O_4_ remains unchanged. As a result, the resistance of the sensor increases, revealing that the materials with a high Co/Sn ratio exhibit p-type sensing behavior (Fig. 4e). In contrast, CO, H_2_S, and NH_3_ gases react with ionic oxygen species on both the SnO_2_ and Co_3_O_4_ surfaces, and electrons move from the gases to the sensor, leading to a decrease in the potential barrier height and n-type sensing behavior (Fig. 4f). This different sensing behavior is caused by the different reactivity of Co_3_O_4_ toward various gases and was further verified by Yin et al.^118^. Similarly, Katoch et al.^119^ demonstrated that SnO_2_-ZnO composites exhibited good selectivity for H_2_ with high sensitivity. This behavior occurs because H atoms can easily adsorb on O sites of ZnO via a strong hybridization between the s-orbitals of H and the p-orbitals of O, which leads to the metallization of ZnO^120,121^.

In summary, we can enhance the sensitivity of type-I sensors by choosing a proper fabrication method, reducing the grain size of the composites, and optimizing the molar ratio of MOS composites. In addition, a thorough understanding of the chemical properties of the sensing materials can further improve the selectivity of the sensors.

### Mechanism of the Type-II Sensor Structure

The type-II sensor structure is another popular sensor structure, and various heteronanostructural materials are available, which consist of one “backbone” nanomaterial and a second or even third nanomaterial. For instance, (1D or 2D materials decorated with nanoparticles, core-shell (C-S), and multilayer heteronanostructure materials are generally used in type-II sensor structures and will be discussed in detail below.

#### Decorated heteronanostructures

**Fig. 5:**
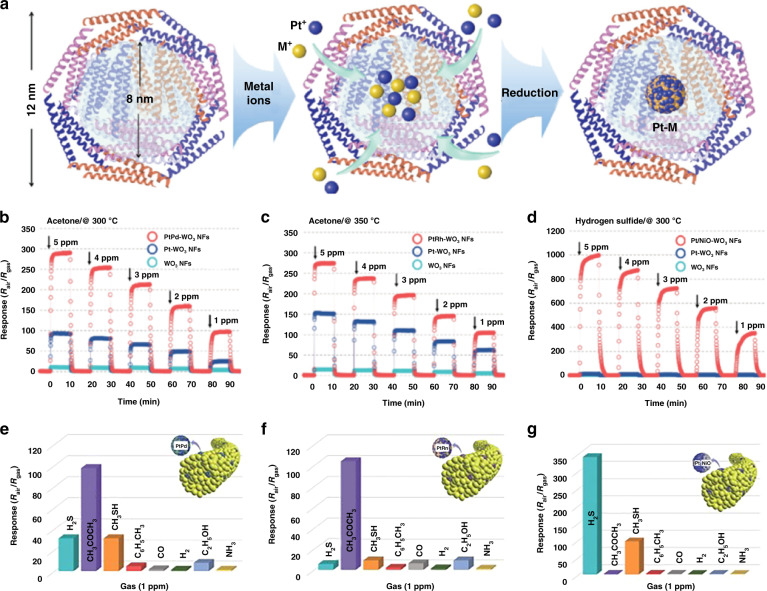
The catalytic effect of the decorated metal NPs. **a** Apoferritin-encapsulating method for preparing PtM-apo NPs (PtPd, PtRh, PtNi); **b**–**d** dynamic gas-sensing properties of pristine WO_3_, PtPd@WO_3_, PtRn@WO_3_, and Pt-NiO@WO_3_ nanofibers; **e**–**g** selectivity property of sensors based on PtPd@WO_3_, PtRn@WO_3_, and Pt-NiO@WO_3_ nanofibers toward 1 ppm interfering gases^126^

For the first kind of heteronanostructural material (decorated heteronanostructures), as demonstrated in Fig. 2b (1), the conduction channel of the sensor is connected by the backbone materials. The modified nanoparticles can provide more reaction sites for adsorption or desorption of gases due to the formation of heterojunctions and can also work as catalysts to enhance the sensing performance^109,122–124^. Yuan et al.^41^ observed that decorating WO_3_ nanowires with CeO_2_ nanodots can provide more adsorption sites at the CeO_2_@WO_3_ heterointerfaces and the CeO_2_ surface and generate more chemisorbed oxygen species to react with acetone. Gunawan et al.^125^. presented an ultrahigh sensitivity acetone sensor based on 1D Au@α-Fe_2_O_3_ and observed that the sensing performance of the sensor is controlled by the activation of O_2_ molecules as the oxygen supply. The presence of Au NPs may act as catalysts to promote the dissociation of oxygen molecules into lattice oxygen for the oxidation of acetone^125^. A similar result was observed by Choi et al.^9^, where Pt catalysts were used to dissociate adsorbed oxygen molecules into ionized oxygen species and enhance the sensing response to acetone. In 2017, the same research group demonstrated that the catalytic effect of bimetallic NPs is much higher than that of single noble nanoparticles, as shown in Fig. 5^126^. Figure 5a shows a schematic graph of the fabrication process for Pt-based bimetallic (PtM) NPs with an average size less than 3 nm using an apoferritin protein cage. Then, electrospinning technology was used to obtain PtM@WO_3_ nanofibers to improve the sensitivity and selectivity toward acetone or H_2_S (Fig. 5b–g). Recently, owing to maximum atom utilization efficiency and tunable electronic structures, single-atom catalysts (SACs) have exhibited superior catalytic performance in the fields of catalysis and gas sensing^127,128^. Shin et al.^129^ used Pt SA-anchored shredded melamine-derived carbon nitride nanosheets (MCN), SnCl_2_, and PVP as chemical sources to prepare Pt@MCN@SnO_2_ fiber-in-tubes for gas sensing. Although the amount of Pt@MCNs was very low (0.13 wt% to 0.68 wt%), Pt@MCN@SnO_2_ showed the highest sensing performance toward formaldehyde gas over other reference samples (pristine SnO_2_, MCN@SnO_2_, and Pt NPs@SnO_2_). This superior sensing performance can be explained by the maximized catalyst atom efficiency of Pt SAs and the minimum coverage of the active sites on SnO_2_^129^.

**Fig. 6:**
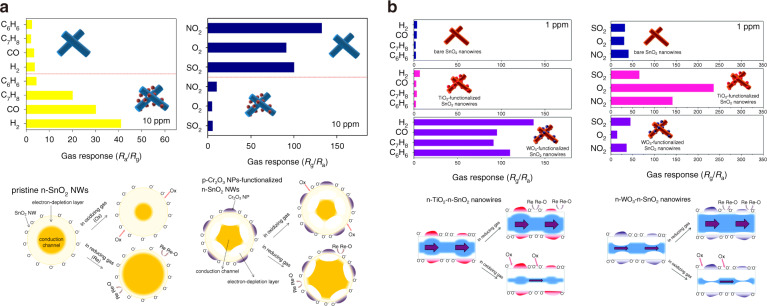
**Radial modulation mechanism caused by decorated MOS NPs**. **a** Summary of gas response to 10 ppm of reducing gases and oxidizing gases based on pure SnO_2_ and Cr_2_O_3_@SnO_2_ nanowires and relevant schematic of sensing mechanisms;^131^ **b** summary of the gas response to 1 ppm reducing gases and oxidizing gases based on pure SnO_2_, TiO_2_@SnO_2_ and WO_3_@SnO_2_ nanorods and relevant schematic of sensing mechanisms^133^

In addition, the formed heterojunctions between the backbone material and the nanoparticles can also efficiently modulate the conduction channel via a radial modulation mechanism to enhance the sensor performance^130–132^. Figure 6a demonstrates the sensing performance of pure SnO_2_ and Cr_2_O_3_@SnO_2_ nanowires to reducing and oxidizing gases and their corresponding sensing mechanisms^131^. Compared with pure SnO_2_ nanowires, the response of the Cr_2_O_3_@SnO_2_ nanowires to the reducing gases is greatly enhanced, whereas it deteriorates to oxidizing gases. These phenomena are closely related to the local suppression of the conduction channel of SnO_2_ nanowires in the radial direction of the formed p-n heterojunction^131^. The resistance of the sensor is simply tuned by changing the width of the EDL on the surface of the pure SnO_2_ nanowires after exposure to the reducing and oxidizing gases^131^. However, for Cr_2_O_3_@SnO_2_ nanowires, the initial EDL of the SnO_2_ nanowires in the air is expanded, and the conduction channel is suppressed compared with that of the pure SnO_2_ nanowires due to the formed heterojunctions. Therefore, when the sensor is exposed to reducing gases, captured electrons are released to the SnO_2_ nanowires, and the EDL dramatically shrinks, resulting in a higher sensitivity than that of the pure SnO_2_ nanowires. In contrast, when switching to oxidizing gases, the expansion of the EDL is limited, which leads to low sensitivity. Similar sensing response results were observed by Choi et al.^133^, where the sensing response of SnO_2_ nanowires decorated with p-type WO_3_ nanoparticles for reducing gases was significantly improved, while an enhanced sensitivity to oxidizing gases was observed for a SnO_2_ sensor decorated with n-type TiO_2_ nanoparticles (Fig. 6b)^133^. This result was mainly due to the different working functions between the SnO_2_ and MOS nanoparticles (TiO_2_ or WO_3_). In p-type (n-type) nanoparticles, the conduction channel of the backbone material (SnO_2_) expands (or shrinks) radially, and then further expansion (or shortening) of the SnO_2_ conduction channel is marginal upon exposure to reducing (or oxidizing) gases (Fig. 6b).

#### Bilayer and multilayer heteronanostructures

In bilayer and multilayer heteronanostructure devices, the conduction channel of the device is dominated by the layer that is directly in contact with the electrodes (generally the bottom layer), and the heterojunctions formed at the contact regions of the two layers can modulate the conductance of the bottom layer. Therefore, once the gases interact with the top layer, they significantly influence the conduction channel of the bottom layer and the resistance of the device^134^. For instance, Kumar et al.^77^ reported the opposite sensing behavior of TiO_2_@NiO and NiO@TiO_2_ bilayer films toward NH_3_. This discrepancy occurs because the conduction channels of the two sensors are dominant in different material layers (NiO and TiO_2_, respectively), and then the changes in the conduction channel in the bottom layer are different^77^.

**Fig. 7:**
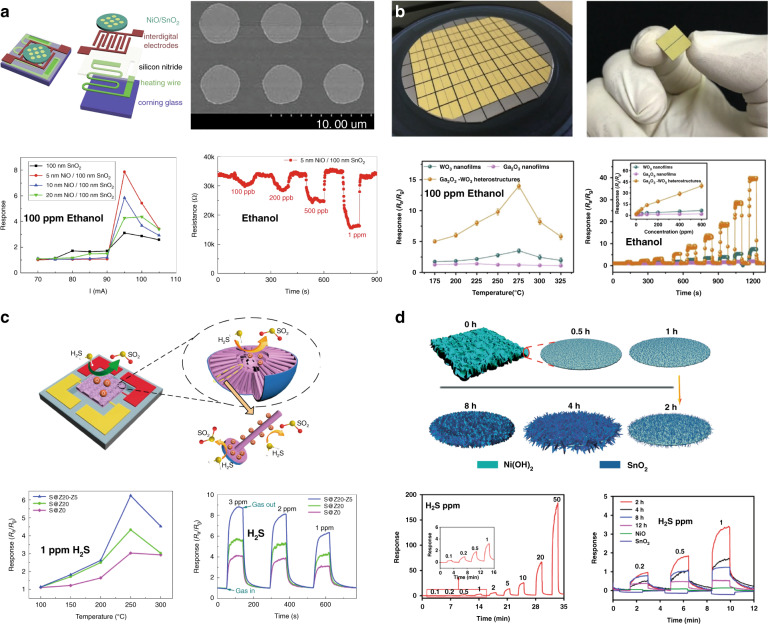
**Gas sensors based on bilayer-MOSs**. **a** NiO@SnO_2_ nanofilms for ethanol sensing;^137^ **b** Ga_2_O_3_@WO_3_ nanofilms for ethanol sensing;^135^ **c** highly ordered bilayer SnO_2_@ZnO hierarchical structures for H_2_S sensing;^139^ **d** bilayer SnO_2_@NiO hierarchical structures for H_2_S sensing^52^

Bilayer or multilayer heteronanostructures are commonly obtained by sputtering, atomic layer deposition (ALD), and spin coating^56,70,134–136^. The thicknesses of the films and the contact areas of the two materials can be well controlled. Figure 7a and b show the NiO@SnO_2_ and Ga_2_O_3_@WO_3_ nanofilms prepared by the sputtering method for ethanol sensing^135,137^. However, these methods usually result in planar films, and the sensitivity of these planar films is lower than that of 3D nanostructured materials due to their low specific surface area and low gas permeation rate. Thus, liquid phase strategies have also been proposed to fabricate bilayer films with different hierarchical structures to increase the sensing performance by increasing the specific surface area^41,52,138^. Zhu et al.^139^ combined sputtering and hydrothermal methods and obtained highly ordered ZnO nanowires on top of SnO_2_ nanobowls (ZnO@SnO_2_ nanowires) for H_2_S sensing (Fig. 7c). Its responsivity to 1 ppm H_2_S is 1.6-fold higher than that of sensors based on the ZnO@SnO_2_ nanofilm, which is prepared by sputtering. Liu et al.^52^ reported a high-performance H_2_S sensor by fabricating SnO_2_@NiO hierarchical nanostructures with an in-situ two-step chemical bath deposition method followed by thermal annealing treatment (Fig. 10d). Compared with conventional SnO_2_@NiO bilayer films, which are prepared by the sputtering method, the sensing performance of the hierarchical SnO_2_@NiO bilayer structure is dramatically enhanced owing to the increase in the specific surface area^52,137^.

#### Core-shell heteronanostructures

In core-shell heteronanostructure (CSHN)-based type-II devices, the sensing mechanism is more complex since the conduction channel is not confined to the inner shell. The fabrication routes and the thicknesses of the shell (*h*_s_) can both determine the location of the conduction channel. For example, the conduction channel is normally confined to the inner core when using bottom-up synthesis approaches, and the structure is similar to bilayer or multilayer device structures (Fig. 2b(3))^123,140–143^. Xu et al.^144^ reported a bottom-up approach to prepare NiO@α-Fe_2_O_3_ and CuO@α-Fe_2_O_3_ CSHNs by depositing a layer of NiO or CuO NPs on top of α-Fe_2_O_3_ nanorods, in which the conduction channel is confined to the core part (α-Fe_2_O_3_ nanorods). Liu et al.^142^ also successfully confined the conduction channel to the core part of TiO_2_@Si CSHNs by drop-casting TiO_2_ onto the as-prepared Si nanowire array. Therefore, its sensing behavior (p-type or n-type) only depends on the semiconducting type of Si nanowires.

**Fig. 8:**
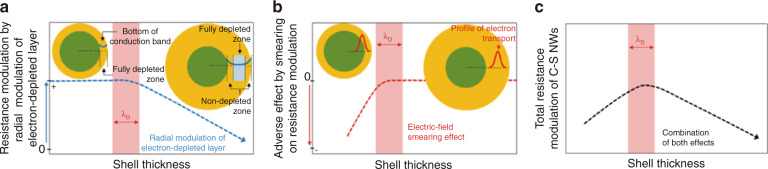
**Conceptual description showing the dual functional sensing mechanism of CS nanowires**. **a** Resistance modulation by radial modulation of the electron-depleted shell layer, **b** adverse effect of smearing on resistance modulation, and **c** total resistance modulation of CS nanowires by a combination of both effects^40^

However, most reported CSHN-based sensors (Fig. 2b(4)) are prepared by transferring synthesized CS material powders onto a chip. In this case, the conduction channel of the sensor is influenced by the thickness of the shell (*h*_s_). Kim’s group studied the effects of *h*_s_ on the gas sensing performance and proposed a possible sensing mechanism^100,112,145–148^. It is believed that two factors contribute to the sensing mechanism of this structure: (1) the radial modulation of the EDL of the shell and (2) the electric field smearing effect (Fig. 8) ^145^. The researchers mentioned that the conduction channel of the carriers is mostly confined to the shell layer when *h*_s_ > λ_D_ of the shell layer^145^. As a result, the resistance modulation of sensors based on CSHNs is mainly dominated by the radial modulation of the EDL of the shell (Fig. 8a). However, when *h*_s_ ≤ λ_D_ of the shell layer, the shell layer becomes fully electron depleted by the adsorbed oxygen species and the formed heterojunction at the CS heterointerface. Therefore, the conduction channel is not only located inside the shell layer but also partially in the core part, especially when *h*_s_ < λ_D_ of the shell layer. In this case, both the fully electron-depleted shell layer and the partially depleted core layer contribute to modulating the resistance of the whole CSHNs, generating an electric-field smearing effect (Fig. 8b). Some other studies use the concept of EDL volume fraction instead of electric field smearing effect to analyze the effect of *h*_s_^100,148^. By taking both contributions into consideration, the overall resistance modulation of the CSHNs reaches the highest when *h*_s_ is comparable with λ_D_ of the shell layer, as shown in Fig. 8c. Therefore, the optimal *h*_s_ of the CSHN may be close to λ_D_ of the shell layer, which is consistent with experimental observations^99,144–146,149^. Several studies have demonstrated that *h*_s_ can also influence the sensing behavior of sensors based on p-n heterojunction CSHNs^40,148^. Lee et al.^148^ and Bai et al.^40^ systematically studied the influence of *h*_s_ on the performance of p-n heterojunction CSHN (*e.g*., TiO_2_@CuO and ZnO@NiO)-based sensors by varying the ALD cycles of the shell layer. As a result, the sensing behavior transits from p-type to n-type with increasing *h*_s_^40,148^. This behavior occurs because at the beginning (with a limited ALD cycle number), the heterostructure can be regarded as decorated heteronanostructures. Thus, the conduction channel is confined to the core layer (p-type MOS), and the sensor shows p-type sensing behavior^40^. By increasing the ALD cycle number, the shell layer (n-type MOS) becomes quasi-continuous and serves as the conduction channel, resulting in n-type sensing behavior^40^. Similar sensing transition behaviors have also been reported on branched p-n heteronanostructures^150,151^. Zhou et al.^150^ studied the sensing behavior of Zn_2_SnO_4_@Mn_3_O_4_ branched heteronanostructures by tuning the content of Zn_2_SnO_4_ on the surface of Mn_3_O_4_ nanowires. The p-type sensing behavior is observed when Zn_2_SnO_4_ seeds form on the surface of Mn_3_O_4_. With a further increase in the content of Zn_2_SnO_4_, the sensor based on Zn_2_SnO_4_@Mn_3_O_4_ branched heteronanostructures switches to n-type sensing behavior.

In summary, the type-II sensor involves many different hierarchical nanostructures, and the sensor performance depends largely on the position of the conduction channel. Therefore, it is crucial to control the location of the conduction channel of the sensor and use a suitable model based on heteronanostructural MOSs to study the enhanced sensing mechanism of the type-II sensor.

### Sensing mechanism of the type-III sensor structure

The type-III sensor structure is not very common, and the conduction channel is based on the formed heterojunction between the two semiconductors, which are separately connected to the two electrodes. The unique device structure is usually obtained via microfabrication techniques, and the sensing mechanism is quite different from those of the previous two types of sensor structures. The *I*–*V* curves of the type-III sensor usually present the typical rectification characteristics due to the formed heterojunctions^48,152,153^. The *I*–*V* curves of the ideal heterojunction could be described by the thermionic emission mechanism of electrons over the heterojunction barrier height^152,154,155^.

1 $$I = I_S\left[ {\exp \left( {\frac{{qV_a}}{{kT}}} \right) - 1} \right] = A\left( {J_n + J_p} \right)$$

where *V*_*a*_ is the bias voltage, *A* is the device area, *k* is the Boltzmann constant, T is the absolute temperature, *q* is the electrical charge of the carrier, and *J*_*n*_ and *J*_*p*_ represent the hole and electron diffusion current densities, respectively. *I*_*S*_ represents the reverse saturation current given by the following equation:^152,154,155^

2 $$I_s = AA^ \ast T^2exp\left( {\frac{{ - qV_{bi}^0}}{{kT}}} \right)$$

where A*is the Richadson constant and $V_{bi}^0$ is the built-in potential.

Therefore, the total current of the p-n heterojunction is determined by both the changes in the carrier concentrations and the heterojunction barrier height, as illustrated by Eqs. (3) and (4) ^156^

3 $$J_p = \frac{{qD_p}}{{L_p}}p_{p0}exp\left( {\frac{{ - qV_{bi}^0}}{{kT}}} \right)\exp \left( {\frac{{ - \Delta E_v}}{{kT}}} \right)\left[ {\exp \left( {\frac{{qV_a}}{{kT}}} \right) - 1} \right]$$

4 $$J_n = \frac{{qD_n}}{{L_n}}n_{n0}exp\left( {\frac{{ - qV_{bi}^0}}{{kT}}} \right)\exp \left( {\frac{{ - \Delta E_c}}{{kT}}} \right)\left[ {\exp \left( {\frac{{qV_a}}{{kT}}} \right) - 1} \right]$$

where *n*_*n*0_ and *p*_*p*0_ are the electron (hole) concentrations in n-type (p-type) MOSs, $V_{bi}^0$ is the built-in potential, *D*_*p*_ (*D*_*n*_) is the diffusion coefficient for the electron (hole), *L*_*n*_ (*L*_*p*_) is the diffusion length for the electron (hole), and Δ*E*_*v*_ (Δ*E*_*c*_) is the energy shift of the valence band (conduction band) at the heterointerface. Although the current density is proportional to the carrier density, it is inversely exponentially correlated with $V_{bi}^0$. Therefore, the overall change in the current density is significantly dependent on the modulation of the heterojunction barrier height.

**Fig. 9:**
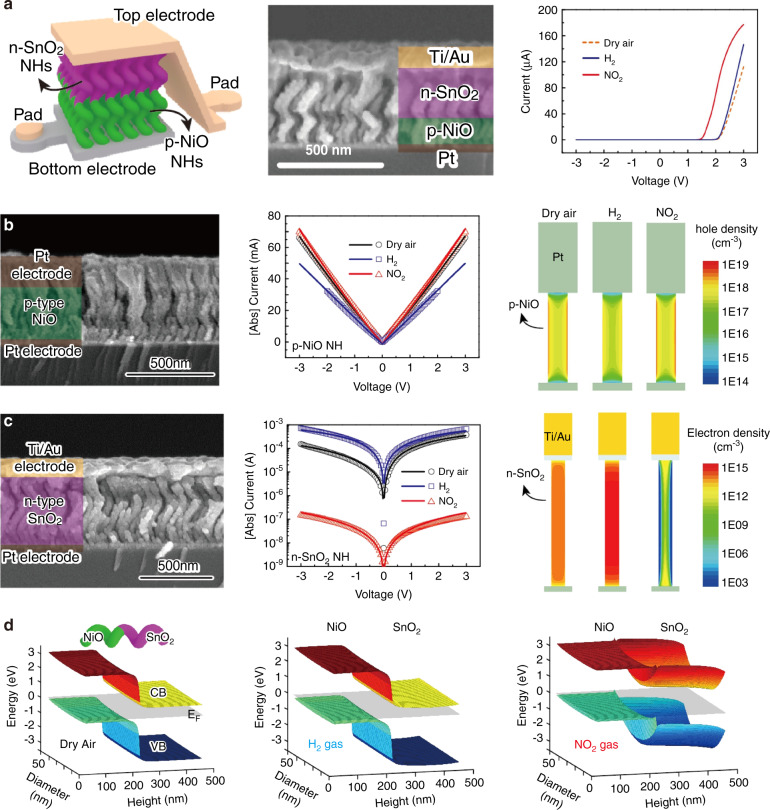
**Sensing mechanism of the gas sensor based on the type-III device structure**. **a** Cross-sectional scanning electron microscopy (SEM) image of the device based on p-NiO/n-SnO_2_ nanohelices and the sensing properties of the p-NiO/n-SnO_2_ nanohelix heterojunction sensor at 200 °C toward H_2_ and NO_2_; **b**, **c** cross-sectional SEM of the devices and simulation results of the devices having **b** the p-NiO layer and **c** the n-SnO_2_ layer. Measured and fitted *I*–*V* characteristics in dry air and after H_2_ and NO_2_ exposure of the **b** p-NiO sensor and **c** n-SnO_2_ sensor. Two-dimensional **b** hole density map in p-NiO and **c** electron map in an n-SnO_2_ layer with color scale simulated by using Sentaurus TCAD software. **d** Results of the simulation showing a three-dimensional map of the p-NiO/n-SnO_2_ in dry air, H_2_ and ambient NO_2_^157^

As discussed above, the construction of heteronanostructural MOS (*e.g*., type-I and type-II devices) can dramatically enhance the sensor performance over that of the individual components. Whereas, for the type-III device, the response of hetero-nanostructure can be either higher than both the components^48,153^ or just higher than only one component^76^ depending on the chemical properties of the materials. Several reports have illustrated that when one of the components is insensitive to target gases, the response of the heteronanostucture is much higher than that of individual components^48,75,76,153^. In this case, the target gas would just interact with the sensitive layer and cause the shift of E_f_ for the sensitive layer as well as the change of heterojunction barrier height^147^. Then, the total current of the device dramatically changes as it is inverse exponentially correlated with the heterojunction barrier height according to Eqs. (3) and (4) ^48,76,153^. However, when both n-type and p-type components are sensitive to target gases, the sensing performance may lie in between. Jose et al.^76^ used the sputtering method to obtain a NiO/SnO_2_ porous thin film-based NO_2_ sensor and found that the sensitivity of the sensor was only higher than that of a NiO-based sensor but lower than that of a SnO_2_-based sensor. This phenomenon is because SnO_2_ and NiO exhibit opposite responses to NO_2_^76^. In addition, as the two components show different sensitivities to gases, they may cause the same trend in sensing oxidizing and reducing gases. For instance, Kwon et al.^157^ proposed a NiO/SnO_2_ p-n heterojunction-based gas sensor via an oblique-angle deposition method, as shown in Fig. 9a. Interestingly, the sensor based on the NiO/SnO_2_ p-n heterojunction showed the same sensing trend to H_2_ and NO_2_ (Fig. 9a). To address this result, Kwon et al.^157^ systematically studied how NO_2_ and H_2_ would change the carrier concentration and modulate the $V_{bi}^0$ of both materials via I-V characterization and computational simulation (Fig. 9 b–d). Figure 9b, c demonstrate the ability of H_2_ and NO_2_ to change the carrier density of the sensor based on p-NiO (*p*_*p*0_) and n-SnO_2_ (*n*_*n*0_), respectively. They show that the *p*_*p*0_ of p-type NiO changed slightly in the NO_2_ environment, while a dramatic change occurred in the H_2_ environment (Fig. 9b). However, *n*_*n*0_ showed the opposite behavior for n-type SnO_2_ (Fig. 9c). Based on these results, the authors concluded that when the sensor based on the NiO/SnO_2_ p-n heterojunction was exposed to H_2_, the increase in *n*_*n*0_ led to an increase in *J*_*n*_, and the slight increase in $V_{bi}^0$ led to a low response (Fig. 9d). After exposure to NO_2_, the large decrease in *n*_*n*0_ in SnO_2_ and the small increase in *p*_*p*0_ in NiO both led to a large decrease in $V_{bi}^0$, which ensured an enhancement of the sensing response (Fig. 9d)^157^. In summary, both changes in the carrier concentrations and $V_{bi}^0$ can lead to the variation of the total current, which further influences the sensing capability.

**Fig. 10:**
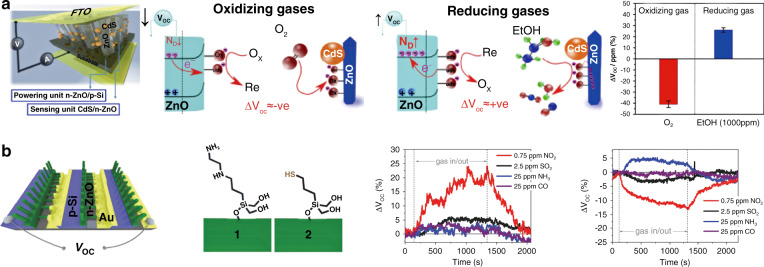
**Photovoltaic self-powered gas sensor based on a type-III device structure**. **a** Self-powered photovoltaic gas sensors based on Si/ZnO@CdS and the self-powered sensing mechanism and sensing responses of the sensor toward oxidizing (O_2_) and reducing (1000 ppm ethanol) gases under solar illumination;^74^ **b** Self-powered photovoltaic gas sensors based on Si/ZnO and the sensing response to different gases after modifying ZnO with amine- and thiol-terminated SAM functionalizations^161^

In addition to the chemical properties of the materials themselves, the Type-III device structure shows the ability to construct a self-powered gas sensor, which cannot be achieved by type-I and type-II devices. The p-n heterojunction diode structure is commonly utilized to construct photovoltaic devices owing to the inherent electric field (BEF) and shows the potential to fabricate photovoltaic room temperature self-powered gas sensors under light illumination^74,158–161^. The BEF at the heterointerface, which results from the difference in Fermi levels of the materials, can also contribute to the separation of electron-hole pairs. The merit of the photovoltaic self-powered gas sensor lies in its low energy consumption, as it can absorb energy from illuminated light and then drive itself or other miniaturized devices without external energy sources. For instance, Tanuma and Sugiyama^162^ fabricated a NiO/ZnO p-n heterojunction as a solar cell to activate a polycrystalline SnO_2_-based CO_2_ sensor. Gad et al.^74^ reported a photovoltaic self-powered gas sensor based on a Si/ZnO@CdS p-n heterojunction, as demonstrated in Fig. 10a. Vertically aligned ZnO nanowires were directly grown on the p-type Si substrate to form a Si/ZnO p-n heterojunction. Then, CdS nanoparticles were decorated on the surface of ZnO nanowires via chemical surface modification. Figure 10a demonstrates the sensing response results of Si/ZnO@CdS to O_2_ and ethanol under self-powered mode. Under light illumination, the open-circuit voltage (*V*_*oc*_), which was induced by the separation of electron-hole pairs under the BEF at the heterointerface of Si/ZnO, increased linearly with the number of connected diodes^74,161^. *V*_*oc*_ can be expressed by Eq. (5) ^156^,

5 $$V_{oc} = \frac{{kT}}{q}\ln \left( {\frac{{N_D^{ZnO}N_A^{Si}}}{{N_i^{ZnO}N_i^{Si}}}} \right)$$

where *N*_*D*_, *N*_*A*_, and *N*_*i*_ are the concentrations of the donor, acceptor, and intrinsic carrier, respectively, and *k*, *T*, and *q* represent the same parameters as in previous equations. When exposure to oxidizing gases, they withdraw electrons from the ZnO nanowires and result in a decrease in $N_D^{ZnO}$ and then *V*_*oc*_. In contrast, the reducing gases lead to an increase in *V*_*oc*_ (Fig. 10a). By decorating ZnO with CdS nanoparticles, the photoexcited electrons in the CdS nanoparticles are injected into the conduction band of ZnO and interact with adsorbed gases, resulting in improved sensing performance^74,160^. Hoffmann et al.^160,161^ reported a similar photovoltaic self-powered gas sensor based on Si/ZnO (Fig. 10b). The sensor can selectively detect NO_2_ by modulating the working function of target gases by functionalizing ZnO nanowires with amine ([3-(2-aminoethylamino) propyl] trimethoxysilane) (amine functionalized-SAM) and thiol ((3-mercapto-propyl)-trimethoxysilane) (thiol functionalized-SAM)) (Fig. 10b)^74,161^.

Therefore, when discussing the sensing mechanism of type-III sensors, it is very important to determine the changes in the heterojunction barrier height and the ability of gases to affect carrier concentrations. In addition, light illumination can produce photogenerated carriers that react with gases, and these carriers are promising for self-powered gas detection.

## Conclusions, future outlook, remaining challenges

As discussed in this literature review, many different MOS heteronanostructures have been fabricated to enhance the performance of sensors. Various keywords (metal oxide composites, core-shell metal oxide, hierarchical metal oxide, and self-powered gas sensor) were searched on the Web of Science database, and the differentiating features (popularity, sensitivity/selectivity, power generation potential, fabrication method, and conduction channel) of the three types of devices are summarized in Table 2. A general concept of how to design high-performance gas sensors has been discussed by analyzing the three key factors proposed by Yamazoe. Researchers have further discussed and classified the enhancement sensing mechanisms of sensors based on MOS heteronanostructures. To understand the influencing factors of gas sensors, various parameters of MOSs (*e.g*., grain size, operation temperatures, density of defects and oxygen vacancies, exposed crystal facet) have been investigated thoroughly. However, the influence of the geometric device structure, which is also critical to the sensing behavior of the sensors, has been ignored and has seldom been discussed. In this review, the basic sensing mechanisms of three typical types of device structures are discussed.

**Table 2:** Comparison of the features of three device structures

Sensor type	Popularity	Sensitivity	Selectivity	Power generation potential	Main fabrication method	Conduction channel
Type-I	g	g	g	p	sol-gel; coprecipitation; hydrothermal; electrospinning; mechanical mixing	Determined by the major MOS
Type-II	e	e	g	p	hydrothermal; sputtering; ALD; spin coating	Normally it is located in the MOS, which is connected with two electrodes; it may also be located in the core layer of the CS structure.
Type-III	p	p	p	e	sputtering; hydrothermal	Channeled by the formed heterojunction

where e means excellent, g means good, and p means poor

The grain size structure, fabrication method, and number of heterojunctions formed by the sensing materials in type-I sensors can greatly influence the sensitivity of the sensors. In addition, the sensing behavior is also influenced by the molar ratio of the components. The type-II device structure (decorated heteronanostructures, bilayer or multilayer films, and CSHNs) is the most popular device structure; it consists of two or more components, and only one of them connects to the electrodes. For this device structure, it is critical to determine the location of the conduction channel and its relative changes when studying the sensing mechanism. As type-II devices involve many different hierarchical heteronanostructures, many different sensing mechanisms have been proposed. In the type-III sensor structure, the conduction channel is dominated by the formed heterojunction at the heterointerface, and the sensing mechanism is quite different. Therefore, it is very important to determine the changes in the heterojunction barrier height of the type-III sensors once exposed to target gases. With this structure, it is possible to fabricate photovoltaic self-powered gas sensors to reduce power consumption. However, there is much progress to be made in studies of self-powered gas sensors since their current fabrication process is rather complicated and the sensitivity is much lower than that of traditional MOS-based chemiresistive gas sensors.

The key advantages of MOS gas sensors with hierarchical heteronanostructures are their fast responses and improved sensitivity. However, several key challenges (*e.g*., high working temperature, long-term stability, poor selectivity and reproducibility, humidity impact, and so on) of MOS gas sensors remain and need to be addressed before they can be used in practical applications. Current MOS gas sensors usually work under high temperatures, which causes high power consumption and impacts the long-term stability of sensors. There are two common approaches to address this problem: (1) the design of low power consumption sensor chips and (2) the development of novel sensing materials that can work at low temperatures or even at room temperature. One method of designing low power consumption sensor chips, one of the method is to minimize the sensor size by fabricating ceramic-based and silicon-based microhotplates^163^. The power consumption of ceramic-based microhotplates is approximately 50–70 mV per sensor, while the power consumption of optimized silicon-based microhotplates can decrease to 2 mW per sensor when operated continuously at 300 °C^163,164^. The development of novel sensing materials is an effective way to reduce the power consumption by decreasing the working temperature and can also improve the stability of the sensor. The thermal stability of MOSs becomes more challenging when the size of MOSs continues to reduce to improve the sensor sensitivity, which results in the signal drift of the sensor^165^. In addition, high temperature promotes the diffusion of materials across the heterointerfaces and form mixed phases, which affects the electronic properties of sensors^166^. Researchers have reported that the optimized operation temperatures of sensors can be reduced by choosing appropriate sensing materials and designing MOS heteronanostructures^167,168^. Seeking a low-temperature method of fabricating highly crystalline MOS heteronanostructures is another promising approach to enhancing stability^168^.

The selectivity of MOS-based sensors is another practical issue because various gases coexist with the target gas, and MOS-based sensors are normally sensitive to more than one gas and usually show cross-sensitivities. Therefore, it is crucial to improve the selectivity of the sensor to a target gas among other gases for real-world applications. Over the past few decades, the selectivity has been partially addressed by constructing gas sensor arrays known as “electronic noses (E-noses)”, and combining computational analysis algorithms such as learning vector quantization (LVQ), principal component analysis (PCA), partial least squares (PLS), and so on^31–34^. Two main factors (the number of sensors, which is greatly related to the kinds of sensing materials, and the computational analysis) are essential for enhancing the ability of the E-nose to distinguish gases^169^. However, it usually requires many complex fabrication processes to increase the sensor number; thus, it is crucial to seek a facile method to enhance the E-nose performance. In addition, modification of the MOS with other materials can also enhance the selectivity of the sensor. For example, one can realize the selective detection of H_2_ using Pd NP-decorated MOSs due to their good catalytic activity^170^. In recent years, some researchers have covered the surface of MOSs with MOFs to improve the sensor selectivity through the size-excusive effect^171,172^. Inspired by this work, the functionalization of materials may address the issue of selectivity in some way. However, much work still needs to be performed on how to choose appropriate materials.

Reproducibility in performance among sensors fabricated under identical conditions and methods is another important requirement of large-scale fabrication and practical applications. Generally, spin- and dip-coating methods are low-cost routes to fabricate gas sensors with high throughput. However, during these processes, the sensing materials tend to aggregate, and the interconnections between the sensing materials and the substrates are weak^68,138,168^. Therefore, the sensitivity and stability of the sensors are significantly influenced, and the performance reproducibility is poor. Other fabrication methods, such as sputtering, ALD, pulsed laser deposition (PLD) and physical vapor deposition (PVD), can directly yield bilayer or multilayer MOS films on patterned silicon or alumina substrates. These techniques can avoid aggregation of the sensing materials and ensure the reproducibility of the sensors and demonstrate the feasibility for large-scale fabrication of planar film-based sensors^173^. However, the sensitivity of these planar films is usually much lower than that of 3D nanostructured materials due to their low specific surface area and low gas permeation rate^41,174^. New strategies for growing MOS heteronanostructures at specific locations on patterned microchips and precisely controlling the size, thickness and morphology of the sensing materials are essential for the low-cost wafer-scale fabrication of sensors with high reproducibility and sensitivity. For instance, Liu et al.^174^ proposed a combined “top-down” and “bottom-up” strategy to manufacture wafer-scale miniaturized gas sensors with high throughput by in-situ growth of Ni(OH)_2_ nanowalls at specific locations of the microhotplate wafer.

In addition, it is important to consider the impact of humidity on the sensor in real-world applications. The water molecules can compete with oxygen molecules for the adsorption site of the sensing materials and influence the responsibility of the sensors to target gases^175^. Similar to oxygen gases, water acts as a molecule via physical adsorption and can also present as hydroxyl radicals or hydroxyl groups in multiple oxidation stations via chemical adsorption^176^. Furthermore, the reliable response of sensors to target gases is a great challenge due to the high level and nonconstant humidity in the environment. Several strategies have been developed to address this issue, such as the gas preconcentration method^177^, humidity compensation and cross-reactive array method^178^ and dehumidification techniques^179,180^. However, these methods are costly and complex and lower the sensor’s sensitivity. Some low-cost strategies have been proposed to suppress the impact of humidity. For example, decorating SnO_2_ with Pd NPs can facilitate the transition of adsorbed oxygen into anionic species, and functionalization of SnO_2_ with materials (*e.g*., NiO and CuO) with a high affinity to water molecules are two possible methods to prevent the humidity dependence of the sensors^181–183^. In addition, the construction of hydrophobic surfaces by introducing hydrophobic materials can also reduce the influence of humidity^36,138,184,185^. However, the design of humidity-resistant gas sensors remains in its early stage, and more advanced strategies are needed to tackle these problems.

In conclusion, improvements in sensing performance (*e.g*., sensitivity, selectivity, low optimal working temperature) have been achieved by the construction of MOS heteronanostructures, and different enhancement sensing mechanisms have been proposed. The geometric device structure must also be taken into consideration when studying the sensing mechanism of a specific sensor. To further improve the performance of gas sensors and address the remaining challenges, exploring novel sensing materials and investigating advanced fabrication strategies are needed in the future. To tune the sensing performance in a controlled way, it is essential to systematically construct relationships between the synthesis methods and the functions of the heteronanostructure for the sensing materials. In addition, the study of the surface reactions and the changes at the heterointerfaces by state-of-the-art characterization techniques can help elucidate their sensing mechanisms and provide guidelines for the design of heteronanostructure material-based sensors. Finally, exploring modern fabrication strategies of sensors may enable the implementation of wafer-scale fabrication of miniaturized gas sensors, which lead to their industrial applications.
